# Effect of Functional Nasal Surgery on Craniofacial Pain: A Prospective Cohort Study

**DOI:** 10.1002/lary.70196

**Published:** 2025-10-21

**Authors:** John R. Craig, Jeewanjot S. Grewal, Anne Grossbauer, Carl Wilson, Robert H. Deeb

**Affiliations:** ^1^ Department of Otolaryngology—Head and Neck Surgery Michigan State University College of Human Medicine Lansing Michigan USA; ^2^ Department of Otolaryngology—Head and Neck Surgery Henry Ford Health Detroit Michigan USA; ^3^ Oakland University William Beaumont School of Medicine Detroit Michigan USA; ^4^ Department of Public Health Sciences Henry Ford Health Detroit Michigan USA

**Keywords:** deviated nasal septum, headache, inferior turbinate reduction, nasal obstruction, septoplasty

## Abstract

**Objectives:**

Functional nasal surgery reliably alleviates nasal obstruction and improves quality of life. However, functional nasal surgery's effect on craniofacial pain (CFP) has been incompletely studied. This study analyzed CFP outcomes following functional nasal surgery.

**Methods:**

A prospective cohort study was conducted with patients who underwent functional nasal surgery for nasal obstruction over 18 months by two surgeons. Nasal Obstruction Symptom Evaluation (NOSE, 0–20) and facial pain scores (FPS, 0–5) were collected preoperatively and postoperatively. NOSE and FPS changes were compared between patients with FPS ≥ 2 versus FPS < 2 (i.e., with vs. without preoperative CFP).

**Results:**

Of 91 patients, mean age was 45.6 years and 62.6% were male. Preoperatively, 36 patients had bothersome CFP, and 12/36 (33.3%) had primary headache disorders. Preoperative mean FPSs were 3.2 and 0.2 for those with versus without preoperative FP, respectively. Mean durations to second and third postoperative visits were 47.7 and 203.1 days, respectively. Across all patients, mean NOSE scores were significantly reduced at each follow‐up (−9.5, *p* < 0.0001). Patients with preoperative CFP achieved significantly greater reductions in FPSs at second (−1.74 vs. +0.24, *p* < 0.0001) and third (−2.20 vs. +0.04, *p* < 0.0001) postoperative visits, and this was not affected by presence of headache disorders or allergic rhinitis (*p* > 0.05). The relative risk (RR) of having FPS ≥ 2 was also significantly reduced at second (RR = 0.44, *p* = 0.0002) and third (RR = 0.33, *p* = 0.003) postoperative visits.

**Conclusion:**

In patients with nasal obstruction and CFP preoperatively, functional nasal surgery led to significant improvements in both nasal obstruction and CFP, and these improvements persisted at about 6 months postoperatively.

**Level of Evidence:**

2.

## Graphical abstract

About 40% of patients presenting for nasal obstruction had craniofacial pain (CPF) preoperatively. In patients with nasal obstruction and CFP preoperatively, functional nasal surgery led to significant improvements in both nasal obstruction and CFP, and these improvements persisted at about 6 months postoperatively.

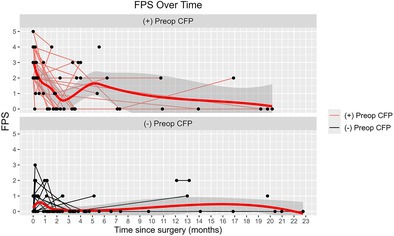

## 1 Introduction

Craniofacial pain (CFP) is a very common complaint in patients presenting to otolaryngologists, and a majority of these patients have primary headache disorders, especially migraine and tension headache [1, 2]. However, much remains to be understood with regard to CFP and how the nasal airway and paranasal sinuses contribute to or cause CFP. Most studies or review articles on nasal obstruction do not discuss concurrent CFP, and very few studies have assessed the effect of functional nasal surgery on CFP in patients with nasal obstruction. One potential reason for the lack of focus on CFP in those with nasal obstruction could be the lack of understanding in how sinonasal pain or pressure is perceived.

The anterior and posterior nasal cavities are lined by respiratory epithelium. Nerve endings reside within or deep to the epithelium and harbor temperature, chemical, and possibly mechanical receptors that respond to a wide variety of such stimuli encountered from the nasal airway [3, 4]. These intraepithelial and subepithelial nerve endings join neighboring nerve endings to represent the nasal mucosal innervation with sensory afferent fibers from the first and second divisions of the trigeminal nerve, and both sympathetic and parasympathetic efferent fibers supplying vascular and glandular elements [4, 5]. Depolarization of trigeminal nerve endings leads to orthodromic sensory responses (e.g., airflow perception and pain), and antidromic responses leading to neuropeptide release from the nerve endings that can elicit mucosal vasodilatory or secretory effects (e.g., nasal obstruction and rhinorrhea) [6]. While the mechanisms behind the perception of nasal airflow remain incompletely studied, mucosal cooling has been shown to be at least partially responsible [7]. Whether a relationship between nasal airflow perception and CFP exists remains to be explored. The purpose of this study was to assess nasal obstruction and CFP outcomes in patients who underwent surgery to alleviate nasal obstruction.

## 2 Materials and Methods

A prospective cohort study was conducted on consecutive adult patients who underwent functional nasal surgery to address primary complaints of nasal obstruction with or without CFP from March 2021 to September 2022 by two surgeons (J.R.C., R.H.D.). Henry Ford Health's Institutional Review Board approved this study protocol, and informed medical consent was obtained from patients. All patients failed at least a one‐month course of topical nasal corticosteroids. They were then offered appropriate nasal airway surgery, which included septoplasty with or without inferior turbinate reduction (ITR) and nasal valve surgery. If patients complained of allergic rhinitis (AR) symptoms, they underwent allergy testing. Exclusion criteria included prior craniofacial trauma, comorbid rhinosinusitis (based on symptoms, endoscopy, or computed tomography), any prior sinonasal surgeries, and those who needed concurrent aesthetic rhinoplasty or sinus surgery.

Demographic data, nasal obstruction sidedness, CFP location, and prior diagnoses of AR and primary headache disorders were recorded. Locations included nasal (external nose), maxillary, frontal, intraorbital, and temporal. Types of nasal surgeries performed were also recorded. Patients were followed for a minimum of 3 months postoperatively. Primary outcome measures were preoperative and postoperative Nasal Obstruction Symptom Evaluation (NOSE, 0–20) and facial pain score (FPS, 0–5) from the 22‐item Sinonasal Outcome Test. Patient cohorts were stratified by whether they did or did not experience preoperative CFP, defined as an FPS ≥ 2. Patients with preoperative FPS < 2 were considered to be without preoperative CFP and acted as an internal control group. Outcomes were collected at second and third postoperative visits, avoiding any confounding from intranasal splints at the first postoperative visit.

Statistical analysis was performed using SAS/STAT v9.3 (Cary, NC, USA) and R software (Vienna, Austria). Demographic and clinical characteristics were first compared between cohorts. Numerical variables were compared via the Wilcoxon ranked sum test and described as means and standard deviations. Categorical variables were described as frequencies and compared via *χ* ^2^ test of independence or Fisher's exact test in the presence of sparse data. FPS and NOSE changes were compared within and between the two cohorts using the Wilcoxon Ranked Sum test. The preoperative CFP cohort was further stratified by whether the patients had premorbid headache disorders, and comparisons were performed to determine whether changes in NOSE and FPS were different between those with versus without headache disorders. Next, the relative risk (RR) of having CFP following nasal surgery over time in those with preoperative CFP was determined via a modified Poisson regression model to account for repeated measurements taken on the same subject over time.

## 3 Results

**FIGURE 1:**
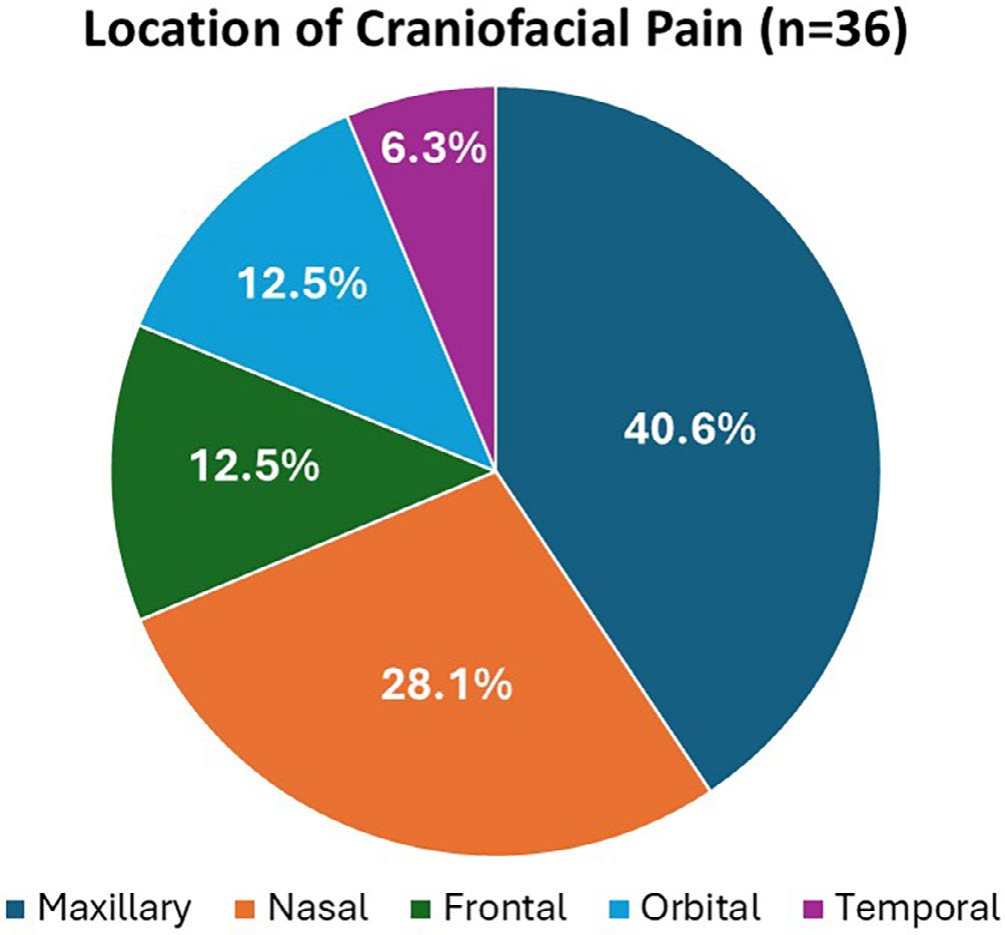
Frequencies of different craniofacial pain locations in patients who presented primarily for nasal obstruction and underwent functional nasal surgery. [Color figure can be viewed in the online issue, which is available at www.laryngoscope.com]

Of 91 patients, mean age was 45.6 ± 16.6 years, and 62.6% were male. Table 1 shows patient demographic and clinical features. About two‐thirds of patients reported bilateral nasal obstruction. Of the 36 patients with CFP preoperatively, 12 (33.3%) had previously diagnosed primary headache disorders, while 24 (66.7%) did not (*p* = 0.045). Figure 1 shows frequencies of preoperative CFP locations, with maxillary (40.6%) and nasal (28.1%) being most common. All patients underwent at least septoplasty, 90/91 (98.9%) had ITR, and 21/91 (23.1%) had nasal valve surgery. Preoperative mean FPSs were 3.2 and 0.2 for those with versus without preoperative CFP (*p* < 0.0001), respectively. Preoperative mean NOSE scores were 15.8 and 13.4 for those with versus without preoperative CFP (*p* = 0.002), respectively.

**TABLE 1:** Demographic and clinical data across all patients, and separated by those with and without preoperative craniofacial pain (CFP).

Variables	Total (*n* = 91)	(−) Preoperative CFP (*n* = 55)	(+) Preoperative CFP (*n* = 36)	*p*
Age (years; mean ± SD)	45.6 (16.58)	46.3 (17.89)	44.6 (14.55)	0.685
Gender [*n* (%)]				
Male	57 (62.6%)	38 (69.1%)	19 (52.8%)	0.116
Female	34 (37.4%)	17 (30.9%)	17 (47.2%)	
Sidedness of obstruction [*n* (%)]				
Left	18 (19.8%)	12 (21.8%)	6 (16.7%)	0.815
Right	13 (14.3%)	8 (14.5%)	5 (13.9%)	
Bilateral	60 (65.9%)	35 (63.6%)	25 (69.4%)	
Primary headache disorder [*n* (%)]	21 (23.1%)	9 (16.4%)	12 (33.3%)	0.060
Allergic rhinitis [*n* (%)]	49 (53.8%)	27 (49.1%)	22 (61.1%)	0.261
Nasal surgery type [*n* (%)]				
Septoplasty	91 (100)	55 (100%)	36 (100)	1.000
Inferior turbinate reduction	90 (98.9%)	55 (100.0%)	35 (97.2%)	0.396
Nasal valve surgery	21 (23.1%)	15 (27.3%)	6 (16.7%)	0.240

Abbreviation: SD, standard deviation.

**FIGURE 2:**
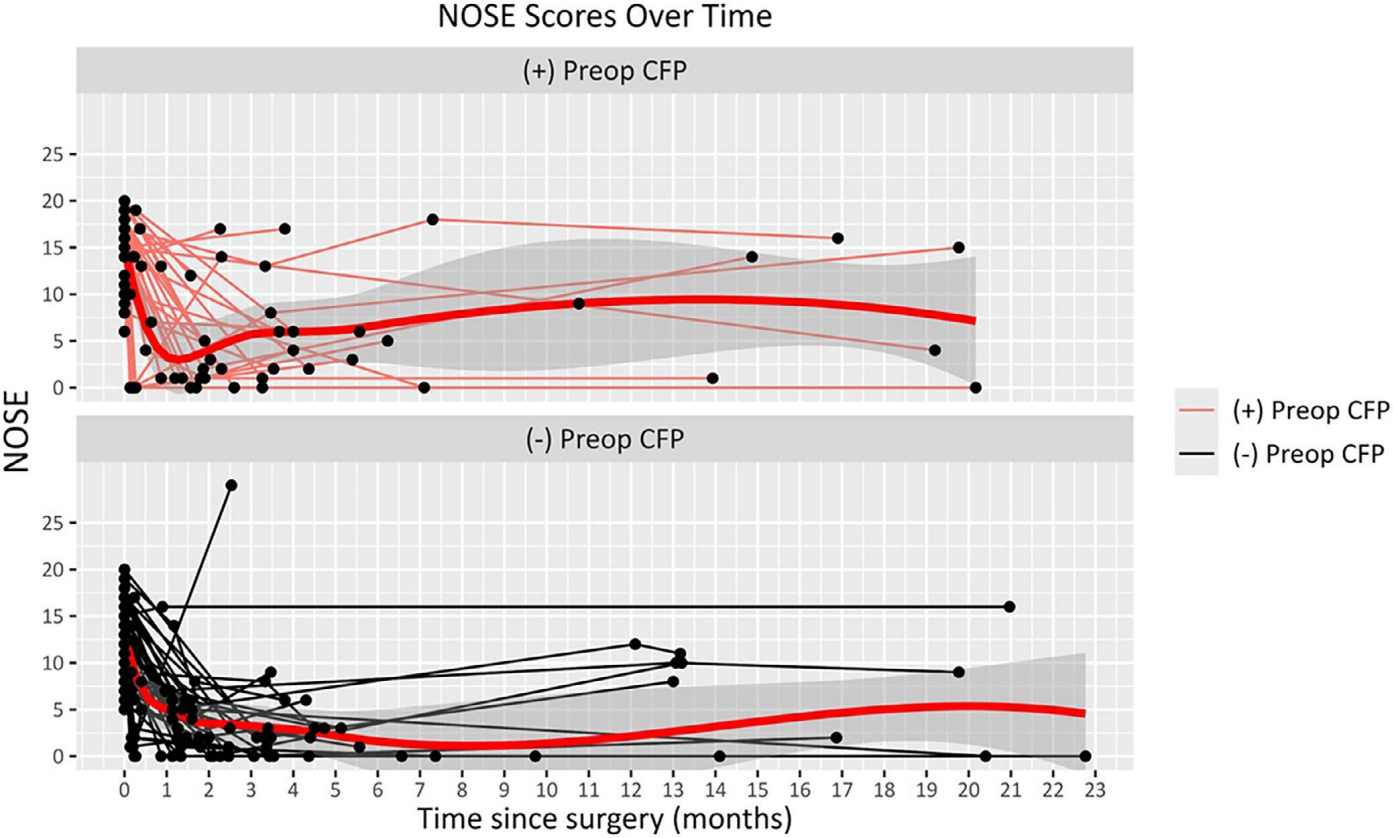
Graph showing the trends in Nasal Obstruction Symptom Evaluation (NOSE) scores over time following functional nasal surgery to address nasal obstruction primarily, in those with versus without preoperative craniofacial pain. [Color figure can be viewed in the online issue, which is available at www.laryngoscope.com]

**FIGURE 3:**
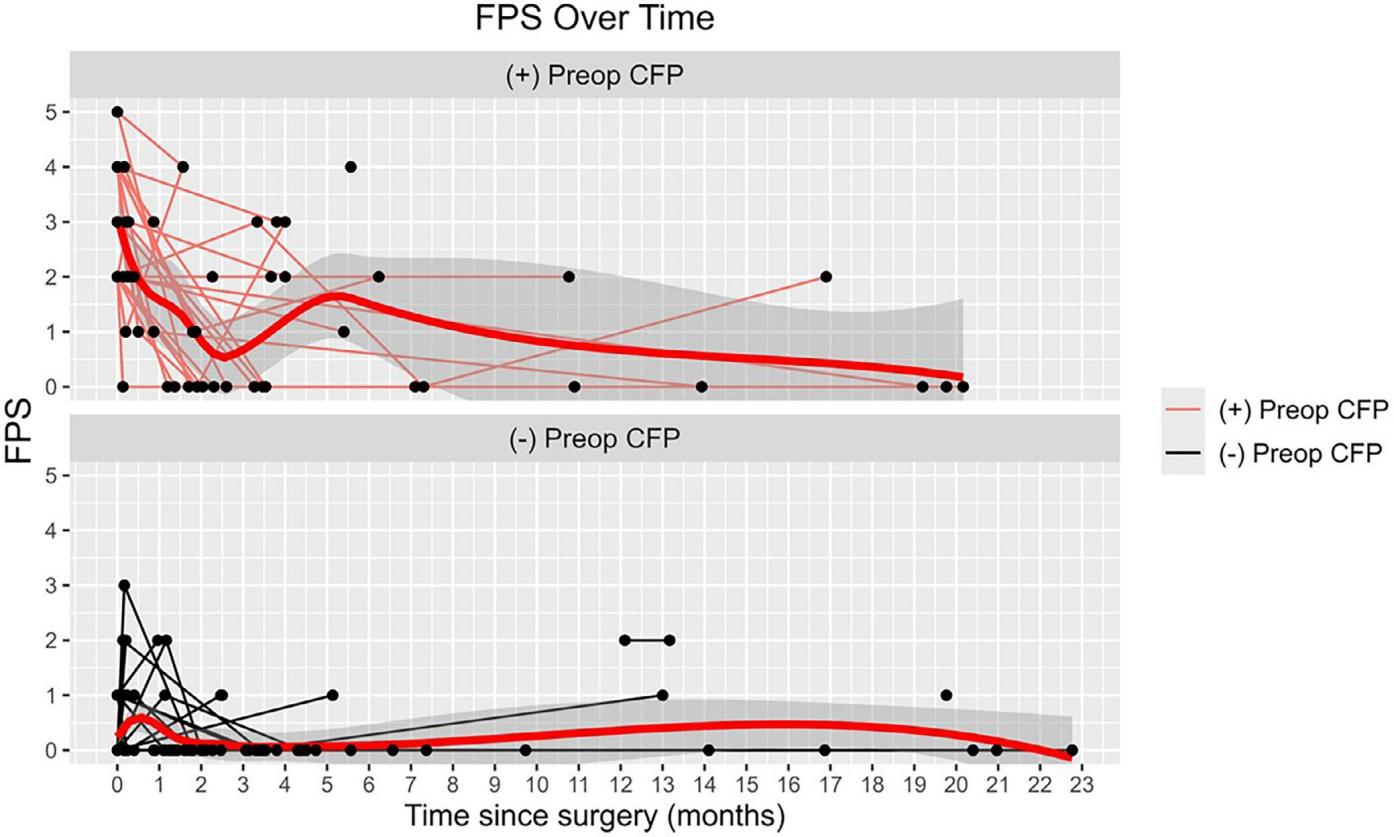
Graph showing the trends in facial pain scores (FPS) over time following functional nasal surgery to address nasal obstruction primarily, in those with versus without preoperative craniofacial pain. [Color figure can be viewed in the online issue, which is available at www.laryngoscope.com]

Mean durations to second and third postoperative visits were 47.7 ± 19.4 and 203.1 ± 71.7 days, respectively, with no significant difference between the cohorts (*p* = 0.840 and *p* = 0.964, respectively). Table 2 shows mean changes in FPS and NOSE at second and third postoperative visits in patients with and without preoperative CFP. Across all patients, mean NOSE scores were significantly reduced at each follow‐up. Among the group with preoperative CFP, there was a significant reduction in FPS postoperatively at each follow‐up. Table 3 shows patients with preoperative CFP achieved significantly greater reductions in FPSs compared to those without preoperative CFP at second (−1.74 vs. +0.24, *p* < 0.0001) and third (−2.20 vs. +0.04, *p* < 0.0001) postoperative visits. There were no differences in changes in NOSE between the two cohorts. Figures 2 and 3 show trends in NOSE scores and FPSs over time in the two cohorts. In patients who had CFP preoperatively, the RR of having FPS ≥ 2 was significantly reduced at second (RR = 0.44, 95% CI: 0.29, 0.68; *p* = 0.0002) and third (RR = 0.33, 95% CI: 0.16, 0.68; *p* = 0.003) postoperative visits. Table 4 shows that the presence of AR and primary headache disorders had no effect on FPS changes in those with preoperative CFP (*p* > 0.05).

**TABLE 2:** Changes in scores for facial pain (FPS) and Nasal Obstruction Symptom Evaluation (NOSE) scores in patients with and without preoperative craniofacial pain (CFP).

Variables in Patients with No Preoperative CFP	Change in Scores for those without preop CFP	*p*	Change in score those with preop CFP	*p*
Change in NOSE				
At second postop	−8.75 (5.49)	**< 0.0001**	−10.52 (7.58)	**< 0.0001**
At third postop	−8.07 (6.56)	**< 0.0001**	−9.81 (6.24)	**< 0.0001**
Change in FPS				
At second postop	+0.24 (0.86)	0.097	−1.74 (1.51)	**< 0.0001**
At third postop	+0.04 (0.46)	1.000	−2.20 (1.70)	**0.001**

*Note*: Bold *p* values represent statistical significance.

**TABLE 3:** Comparison of changes in facial pain scores (FPS) and Nasal Obstruction Symptom Evaluation (NOSE) scores from preoperatively to postoperatively in patients with versus without preoperative craniofacial pain (CFP).

Variables	(−) Preoperative CFP (*n* = 55)	(+) Preoperative CFP (*n* = 36)	*p*
Change in NOSE			
Second postop	−8.75 (5.49)	−10.52 (7.58)	0.126
Third postop	−8.07 (6.56)	−9.81 (6.24)	0.470
Change in FPS			
Second postop	+0.24 (0.86)	−1.74 (1.51)	**< 0.0001**
Third postop	+0.04 (0.46)	−2.20 (1.70)	**< 0.0001**

*Note*: Bold *p* values represent statistical significance.

**TABLE 4:** Comparison of changes in facial pain scores (FPS) from preoperatively to postoperatively in patients with preoperative craniofacial pain (CFP), stratified by whether they had either primary headache disorders or allergic rhinitis. Neither headaches nor allergic rhinitis affected reductions in FPS postoperatively.

Variables	(−) Primary headache disorder (*n* = 24)	(+) Primary headache Disorder (*n* = 12)	*p*	(−) Allergic rhinitis (*n* = 14)	(+) Allergic rhinitis (*n* = 22)	*p*
Change in FPS						
Second postop	−1.6 (1.66)	−1.9 (1.29)	0.489	−2.1 (1.38)	−1.4 (1.60)	0.215
Third postop	−2.3 (1.71)	−2.0 (2.00)	0.825	−1.4 (2.19)	−2.6 (1.35)	0.287

## 4 Discussion

While most patients presenting to otolaryngologists for CFP, especially “sinus headache,” will not have infectious or inflammatory rhinosinusitis [1, 2, 8], significantly fewer studies have explored whether CFP can be related to nasal airway obstruction. The nasal mucosa encounters a myriad of stimuli that could potentially cause CFP, via both neural pathways with the central nervous system and local nerve reflexes. The nasal mucosa is densely innervated by trigeminal and autonomic nerves [5, 9]. These nerves extend free nerve endings with various membrane‐spanning ligand‐gated ion channels that can be activated by numerous chemical, thermal, and mechanical stimuli [4]. Whether nasal airflow restriction can directly or indirectly lead to CFP generation has been incompletely studied, but prior literature would suggest a possible relationship.

First, studies on patients presenting primarily for sinus headache and CFP have reported high rates of nasal obstruction in 43%–72% of patients [1, 2, 10], often speculated to stem from autonomic imbalance or other neural mechanisms, though without clear experimental validation [2, 11]. On the other hand, studies on patients presenting primarily for nasal obstruction have reported about a 23%–58% rate of concurrent headaches or CFP [12, 13]. The current study also showed that about 40% of the nasal obstruction patients had some degree of bothersome CFP preoperatively. Of these patients, only 33.3% had been diagnosed with a primary headache disorder, making it possible that their preoperative CFP was due to rhinogenic factors rather than headache mechanisms. Based on the aforementioned and current studies, CFP may be caused by both primary headache mechanisms and nasal airflow aberrations. On the contrary, some or all the CFP patients without headache disorders could have had undiagnosed headache conditions. Future studies should explore whether CFP mechanisms and response to nasal airway surgery differ in patients with versus without headache disorders.

Only a few prior studies have assessed CFP outcomes after nasal airway surgery while excluding patients with sinusitis or isolated CFP. One prospective study in 1986 in Denmark included 444 patients who underwent septorhinoplasty with or without ITR for nasal obstruction, and 157 had chronic headaches. Based on surveys, 70.3% of patients experienced complete headache resolution, and another 11.0% experienced improvement at 12 months postoperatively. They showed a significant association between improvements in nasal obstruction and CFP [14]. They then followed 70 of their patients with examination and surveys at 5–8 years postoperatively, and while the overall cure and improvement rates remained stable, there were some changes across individual patients in both improvement and worsening [15]. Another study included 55 patients who underwent septoplasty alone in the setting of symptomatic nasal obstruction with chronic headaches. After 4–48 months of postoperative follow‐up, 63.6% achieved headache relief, with more likely improvement with frontal headaches and pressure‐like CFP [12]. Regarding CFP locations in the setting of nasal obstruction, the previously mentioned studies and others have shown frontal to be the most common CFP location in 53%–87% of patients, though significant variability in CFP location and sidedness has been shown in each study [12, 14, 16].

The current study built on these prior studies with clear inclusion and exclusion criteria, validated NOSE scores to compare with subjective FPSs, and conducted a statistical evaluation of the changes in these scores. One interesting and novel finding in this study was that those with preoperative CFP had statistically significantly higher preoperative NOSE scores compared to those without preoperative CFP. Whether this difference is clinically significant would require further study, but this strengthens the possibility that restricted nasal airflow can be associated with CFP. Another difference when comparing this study to prior studies was that patients with preoperative CFP had maxillary and nasal pain most commonly, as opposed to frontal. This study also showed that the improvements in both CFP and nasal obstruction were not related to AR or headache disorders. Taken together, prior studies and the current study show a preponderance of improvement in CFP following nasal airway surgery, and the pain improvement could be related to improved nasal airflow.

How might improved nasal airflow lead to reduced CFP? Answers remain speculative, but there are several possibilities. First, a placebo effect is possible, especially since no control group was available for comparison in this study. For example, even following vertebroplasty for chronic back pain, high rates of symptomatic pain improvement have been demonstrated in placebo groups [17, 18]. This was potentially mitigated to some degree in the current study by patients' primary complaints being nasal obstruction. All patients were counseled that CFP resolution following nasal airway surgery would be unpredictable and that the primary goal of the surgery was to improve nasal airflow. That said, some patients could have held out hope for pain resolution, and this could have promoted a placebo effect. Another potential explanation for CFP improvement could be intranasal nerve injury incurred during nasal surgery. Of note, this injury could be temporary, and a mean 6‐month follow‐up could have prevented detection of eventual pain recurrence upon nerve regeneration in this study. On the contrary, perhaps intranasal nerve injury during nasal surgery leads to long‐term decreases in sensory activation (orthodromic pain) and neurogenic inflammation (antidromic responses like obstruction and rhinorrhea associated with pain).

Another potential mechanism stems from transient receptor potential (TRP) channels on mucosal nerve endings. TRPs are ion channels that act as intranasal sensors to detect a wide variety of thermal and chemical stimuli. TRPM8 is one such channel activated by menthol and cold temperatures < 25°C [4], and activation leads to a sensation of increased nasal airflow. TRPA1 is activated at colder temperatures < 17°C to induce pain (noxious cold) [4]. While multiple studies have shown that nasal obstruction is more a function of nasal mucosal cooling than nasal airway resistance [7, 19], these studies have not explored a possible mechanistic connection between nasal airflow and CFP. Notably, multiple studies on humans and animals have shown that menthol‐induced TRPM8 stimulation produces analgesia. It is theoretically possible that by improving mucosal cooling through nasal airway surgery, TRPM8 is more effectively activated, invoking a state of relative analgesia [20]. However, if TRPM8 is overstimulated by cold temperatures for extended durations, it can lead to cold‐induced pain, which might explain some patients' pain in the postoperative period. There are other TRPs as well, and whether these TRP receptors contribute to CFP resolution following nasal airway surgery will require further research.

In addition to the potential for thermal and chemical stimuli to govern CFP in the setting of nasal obstruction, it would be reasonable to consider mechanoreceptors detecting pressure or resistance changes within the nasal mucosa. Other areas of the body, like the spinal cord, bone, and bowel, contain mechanoreceptors to detect pressure changes, and activation of these receptors can then elicit pain [21, 22]. Significantly less research has been performed regarding sinonasal mechanoreceptors. Frasnelli et al. showed that the posterior nasal cavity mucosa was more sensitive to mechanical stimuli (air puffs) compared to the anterior nasal mucosa, whereas the anterior nasal cavity was more sensitive to chemical stimuli (carbon dioxide) [3]. More research is needed to explore whether different chemical and mechanical stimuli can lead to CFP, ideally with subjective and objective neurophysiologic testing.

Regarding other physiological mechanisms, considering 97% of patients in this study underwent ITR, it is possible that ITR improved patients' CFP through a reduction in vascular engorgement. Evidence on turbinate enlargement being linked to CFP is limited, but was suggested by a study by McAuliffe et al. in 1950 [23]. Some authors have also suggested mucosal contact points as a cause of CFP, though evidence has been mixed. Patel et al. published an evidence‐based review on this topic in 2015, and highlighted that there was a preponderance of benefit in small studies in highly select patients with contact points, headaches, and normal CT scans. However, they also emphasized that long‐term follow‐up was missing from most studies, and no controlled studies had been performed [24]. Of note, their review only covered primary headache complaints, and did not discuss the potential of alleviating CFP through nasal airway surgery to address nasal obstruction. Another systematic review by Harrison and Jones showed that mucosal contact point presence was a poor predictor of CFP, and surgical intervention rarely resulted in complete pain resolution, suggesting that contact points are unlikely to cause CFP [25]. Whether contact points contributed to the outcomes of the current study cannot be determined, and would require further study.

Several limitations should be considered with this study. This was a single‐center study with a relatively small sample size, and follow‐up duration was limited. It is possible that pain could recur even years after surgery, so this study should not necessarily imply that nasal airway surgery will lead to long‐term CFP cessation. Another limitation was the use of subjective measures of nasal obstruction and CFP, without objective airflow or neurophysiological measures. Additionally, CFP sidedness and features (e.g., severity, duration, or quality like burning or stabbing) were not recorded, and future studies could explore whether specific pain features are predictive of response to nasal airway surgery. CFP was also not distinguished between those with versus without headache disorders, so whether CFP was caused by or distinct from one's underlying headache disorder could not be determined. Finally, there was no control group of preoperative CFP patients who did not undergo nasal airway surgery. This prevented the ability to establish causality between surgery and CFP improvement. However, this would be challenging or impossible due to the ethical concerns of withholding indicated nasal airway surgery in patients with symptomatic nasal obstruction.

## 5 Conclusion

In patients with nasal obstruction and CFP preoperatively, functional nasal surgery led to significant improvements in both nasal obstruction and CFP, and these improvements persisted at about 6 months postoperatively.

## Conflicts of Interest

John R. Craig: Research consultant for Aerin Medical Inc. The other authors declare no conflicts of interest.

## Data Availability

The data that support the findings of this study are available on request from the corresponding author. The data are not publicly available due to privacy or ethical restrictions.
